# A quasi-experimental study of the effects of an integrated care intervention for the frail elderly on informal caregivers’ satisfaction with care and support

**DOI:** 10.1186/1472-6963-14-140

**Published:** 2014-03-29

**Authors:** Benjamin Janse, Robbert Huijsman, Isabelle Natalina Fabbricotti

**Affiliations:** 1 Erasmus University Rotterdam, Institute of Health Policy and Management, P.O. Box 1738, 3000, DR Rotterdam, the Netherlands

**Keywords:** Integrated care, Frail elderly, Informal caregivers, Satisfaction, Care and support, Services

## Abstract

**Background:**

This study explored the effects of an integrated care model for the frail elderly on informal caregivers’ satisfaction with care and support services.

**Methods:**

A 62-item instrument was developed and deployed in an evaluative before/after study using a quasi-experimental design and enrolling a control group. The definitive study population (n = 63) consisted mainly of female informal caregivers who did not live with the care recipient. Analysis of separate items involved group comparisons, using paired and unpaired tests, and regression analyses, with baseline measurements, control variables (sex, age and living together with care recipient) and the intervention as independent variables. Subsequently, the underlying factor structure of the theoretical dimensions was investigated using primary component analysis. Group comparisons and regression analyses were performed on the resulting scales.

**Results:**

Satisfaction with the degree to which care was provided according to the need for care of the recipients increased, while satisfaction with the degree to which professionals provided help with administrative tasks, the understandability of the information provided and the degree to which informal caregivers knew which professionals to call, decreased. Primary component analysis yielded 6 scales for satisfaction with care and 5 scales for satisfaction with caregiver support, with sufficient reliability.

**Conclusions:**

The results suggest that expectations regarding the effects of integrated care on informal caregiver satisfaction may not be realistic. However, the results must be seen in light of the small sample size and should therefore be considered as preliminary. Nonetheless, this study provides guidance for further research and integrated care interventions involving informal caregivers.

**Trial registration:**

Current Controlled Trials ISRCTN05748494. Date of registration: 14/03/2013.

## Background

Informal caregivers of frail elderly people often perform a substantial number of care tasks over a prolonged period of time [1]. By definition, informal care is non-professional and unpaid and is provided by family members, partners or close friends [2]. Frail elderly people suffer from age-related problems in different domains of daily functioning, such as physical, psychological, and social problems [3]. Their informal caregivers must frequently interact with the healthcare system to obtain the information, services, and equipment needed to counter such problems [4]. However, many informal caregivers experience the healthcare system as fragmented, rigid and difficult to access [5]. In addition, while it is evident that providing informal care can lead to substantial deteriorations in health and quality of life [6-8], support services for informal caregivers are still often inadequate [9,10]. This lack of explicit attention to informal caregivers denotes a serious gap in healthcare [5]. Consequently, many authors have called for more consideration of informal caregivers’ needs for attention and support [7,11-13], as well as greater insight into their perceptions and satisfaction with such services [14].

As a result, increased attention has been paid to the involvement and support of informal caregivers of frail elderly patients [15-17]. In this context, particular interest has been given to including informal caregivers in integrated care arrangements [10,18]. Integrated care is defined here as a ‘coherent set of methods and models on the funding and on the administrative, organizational, service delivery and clinical levels designed to create connectivity, alignment, and collaboration within and between the cure and care sectors’ [19]. Integrated care has been proposed to increase the coherence, continuity and quality of elderly care [19,20] and to provide more adequate and effective support for informal caregivers [9]. The proactive nature of integrated care is assumed to increase the likelihood of a timely recognition of unmet needs of both the care recipient and informal caregiver [21]. In addition, as it includes coordination mechanisms, such as case management, integrated care is believed to benefit informal caregivers by linking them to adequate formal services [22]. Such characteristics are assumed to increase the satisfaction of informal caregivers with the care provided to the care recipients, as well as with the way these services support themselves as caregivers [18,23].

Although the call for greater attention to the informal caregiver dates back as far as 1990 (e.g., [12]), little is known regarding caregivers’ satisfaction with services [5]. Moreover, despite the substantial number of studies produced in recent years regarding integrated care arrangements, the role of informal caregivers therein has largely been neglected [9]. Consequently, a gap exists in the literature regarding the effects of integrated care on informal caregivers’ satisfaction [23]. Some studies have reported outcomes regarding this subject and have confirmed that integrated care indeed increases informal caregivers’ satisfaction with services [15,18,24]. However, the interventions and their subsequent evaluations were aimed primarily at improving care for elderly patients. Although these interventions acknowledged informal caregivers by involving them to some degree in the care process, they were regarded more as partners in care than as potential individuals in need of care and support. Satisfaction assessment in these studies was therefore related only to the care provided to the care recipients and was not related to the care and support provided to the informal caregivers themselves. To our knowledge, there have been no studies that have investigated the effects of integrated care on informal caregiver satisfaction with the care received by the care recipient in combination with their satisfaction with the care and support they personally received. This study therefore describes the construction of a caregiver satisfaction instrument and its use in the evaluation of informal caregiver satisfaction with a specific integrated care intervention.

### Study aim

The aim of the current study was to investigate the effects of integrated care on informal caregivers’ satisfaction with the care received by care recipients and on the satisfaction with the care and support the caregivers’ themselves received. To this end, a specific integrated care intervention aimed at frail elderly patients was evaluated. This intervention, the Walcheren Integrated Care Model (WICM), has been implemented in the Walcheren region in the southwest of the Netherlands. The research question guiding this study was: What are the effects of the Walcheren Integrated Care Model on the satisfaction of informal caregivers with care and support services?

### Intervention

**Figure 1:**
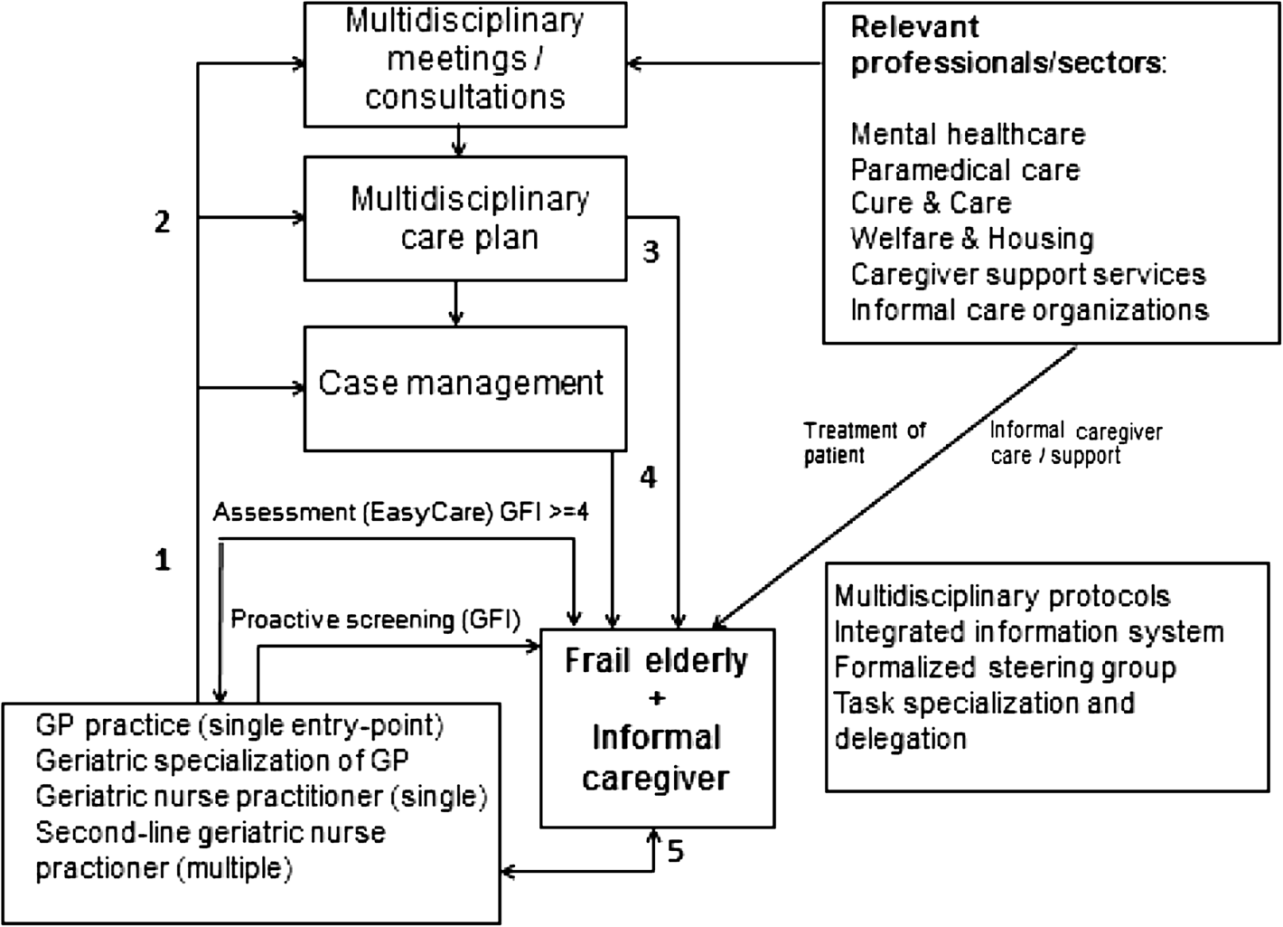
The walcheren integrated care model.

The study protocol and an extensive description of the intervention have been described elsewhere [21]. The Walcheren Integrated Care Model targets independently living frail elderly individuals (living in their own homes or in some form of assisted living) and their informal caregivers. It contains several evidence-based components: a screening tool for the detection of frailty in the elderly, a single entry point, an evidence-based comprehensive need assessment tool, a multidisciplinary individualized service plan, case management, multidisciplinary team consultation and meetings, protocol-led care assignment, a steering group, task specialization and delegation, and an Integrated Information System (Figure 1). The model was implemented in the Walcheren region in the southwest of the Netherlands in 2010 by the regional cooperative of general care practices and was funded by the regional healthcare insurer. Planning, design and funding of the WICM aimed to provide sustainable integrated care to frail elderly patients beyond the period of evaluation of the current study (12 months).

Usual care for frail elderly patients in the Netherlands can be described as reactive and mono-disciplinary. General practitioners (GPs) are generally only consulted on the initiative of their patients. Patients have access to a number of care and curative services through referral of their GP, while other services, such as home-care and personal care services are arranged by municipalities [25]. Complex care services (e.g., injections, decubitus care) are available after formal approval by an assessment agency. Care as usual does not include multidisciplinary coordination or cooperation between these professionals, organizations and professionals. In contrast, the WICM has an outspoken proactive and multidisciplinary character, with the GP and primary care practice (PCP) functioning as a single-entry point for all care requests. This proactive character of the model adds a strong preventive element to the process.

The involvement of informal caregivers begins after screening and needs assessment of the care recipient during a visit by the case manager (step 1 in Figure 1). During this phase, the informal caregiver’s needs for support and guidance are identified. Available services for informal caregivers normally include respite care or other forms of relief, as well as psychosocial interventions such as education and training or (group) counseling. In the WICM, the case manager provides the informal caregiver with relevant information, advice and suggestions regarding these services. If needed, the informal caregiver is brought into contact with relevant organizations or professionals. In addition, case managers may also provide practical advice (e.g., how to make care tasks less burdensome) or emotional support, depending on the informal caregiver’s needs.

The case manager draws up care goals containing proposals for needed assistance and support in consultation with the care recipient and informal caregiver, ensuring the explicitly involved of the informal caregiver in the planning and subsequent provision of care (step 2 in Figure 1). The care plan is then discussed in a multidisciplinary meeting, which is attended by the general practitioner (GP), the case manager and any other care professionals relevant to the care process of the patient. During this meeting, tasks are assigned to the relevant professional based on multidisciplinary protocols. After final approval by professionals, the patient and the informal caregiver (step 3 in Figure 1), the care plan is incorporated into an Integrated Information System accessible to all relevant professionals. The case manager coordinates all care provision and periodically evaluates the care plan with the care recipient and informal caregiver (visits/telephone) to ensure adequate monitoring of their needs (step 4 in Figure 1). The frequency of periodic evaluations ranges from once a month to once a year, depending on the specific situation and needs of the care recipient and informal caregiver. In addition, patients and informal caregivers can contact their case manager at any time in between evaluations (step 5 in Figure 1).

### Theory

Rather than being a global construct, satisfaction with care is generally regarded as a multidimensional construct [26]. Therefore, the literature on informal caregiver satisfaction with various types of care and support services was reviewed to determine the potential dimensions.

The first dimension of satisfaction with care is information provision. Informal caregivers greatly value clearly formulated care arrangements, adherence to these arrangements by professionals and adequate coordination [27-29]. In a study on satisfaction with case management, informal caregivers indicated that such clarity should extend to all information that is provided [27], including information regarding when, how and by whom care is provided, as well as information regarding the care recipient’s disease trajectory and available care and support services [15,27-29]. Informal caregivers also need information to address patients’ care and treatment demands themselves. Such information must be relevant, clear and understandable [4]. Furthermore, information provision should be directed toward relieving informal caregivers’ uncertainties and managing their expectations [30,31]. Informal caregivers appreciate timely and ongoing communication, especially when changes have been made in care provision arrangements [27,32,33]. Care professionals should take sufficient time to convey such information adequately [4]. In addition, it is appreciated when communication occurs through one central source (e.g., the case manager or GP) [34].

A second dimension is associated with the feelings of control and involvement of informal caregivers. Satisfaction of informal caregivers depends on the degree of control over when, how and by whom care is delivered [27]. Several studies have emphasized that it is necessary to involve informal caregivers actively in all aspects of care provision and care planning [28,29,34]. To foster informal caregiver satisfaction, care professionals should therefore collaborate with both the care recipient and his or her caregiver in the development and implementation of care plans [27,35]. Feeling part of a team and being treated as an equal by care professionals can contribute to informal caregiver satisfaction [34]. This sense of control also applies to informal caregivers knowing what is expected from them in terms of their roles and care responsibilities [27]. In this context, satisfaction can increase by discussing and determining the appropriate tasks of care professionals and informal caregivers [36].

A third dimension is best described as client-centeredness and professionalism. Client-centeredness is expressed by attentiveness to the needs, abilities and specific circumstances of care recipients and their informal caregivers [34]. It also involves care professionals being informed about the likes, dislikes and routines of the care recipients [5,27]. Professionalism is the manner in which care professionals approach and treat informal caregivers and care recipients. Empathy, supportiveness, compassion [5,34] and sensitivity [37] have been reported to be vital characteristics of care professionals that contribute to informal caregiver satisfaction. Informal caregivers want to be treated with respect and their care recipients to be treated with dignity [35].

The fourth and final dimension involves the quality and amount of care. Care and support services that are flexible and compatible with the needs of both the care recipient and informal caregiver have been reported to contribute to informal caregiver satisfaction [5,15,27-29,38]. Informal caregivers see the monitoring of the quality of care as an important part of their role [39]. Therefore, dissatisfaction can occur when there is a lack of adequate services, if the quality of services is perceived as insufficient or if there are considerable limitations to accessibility (e.g., long waiting lists) [27,34,40]. Dissatisfaction can also occur if many different care professionals are involved, especially when their composition is constantly changing [34]. In terms of support services for informal caregivers, it has been noted that the provision of assurance, advice and emotional support is important [15,35]. In addition, the provision of one-on-one professional guidance and ongoing case coordination is highly valued [5,15].

## Methods

### Study design and participants

The design of this study was quasi-experimental and included before and after measurements and a control group. Baseline measurements (T0) were obtained prior to the intervention. Follow-up measurements (T1) were obtained 12 months after T0. The study protocol (protocol number MEC-2013-058) was reviewed by the medical ethics committee of the Erasmus Medical Centre Rotterdam in the Netherlands. The committee waived further examination, as the Medical Research Involving Subjects Act did not apply. The study subjects were informal caregivers of frail elderly patients in 8 primary care practices (PCPs) in the Walcheren region. Prior to the intervention, these patients were asked whether they received informal care, and if so from whom. Inclusion of informal caregivers as subjects in the current study was only possible if the patients received a type of formal care. This criterion was required as informal caregivers would have to be able to assess formal care services. Written informed consent was obtained from all of the participating elderly patients and their informal caregivers.

Three PCPs (6 GPs) provided care according to the WICM and constituted the experimental group. The control group consisted of 5 PCPs (6 GPs) delivering care as usual. As patients (and their informal caregivers) that participated in the WICM were approached by the researchers, control practices were blind regarding the participation of patients and informal caregivers as control subjects. Thus, the possibility of patients being treated differently was ruled out.

### Data collection

Trained interviewers visited the participating frail patients at home. All of the interviewers lived in the region and had a background in elderly care. If the informal caregiver was present, data were collected using the questionnaire by face-to-face interview. If not, contact information was obtained from the elderly patient and the informal caregiver was approached by mail or telephone.

### Questionnaire

A literature search yielded no validated instrument to measure informal caregiver satisfaction applicable to the specific context of the current study. Other studies have used the Client Satisfaction Questionnaire (CSQ-8), a validated instrument to measure global patient satisfaction with services [41], and have adapted it for informal caregivers [15,18,42]. However, the SCQ-8 was not deemed appropriate for the current study because of its global character. The authors decided that this global character would lack the sensitivity to the different components of (integrated) care services. Other instruments for assessing informal caregiver satisfaction exist, but they have only been validated for other types of care, such as hospital stroke care (e.g., [43,44]).

A questionnaire was thus constructed by the researchers based on the main dimensions derived from the literature. From these dimensions and from existing questionnaires [44,45], operationalizations were made (see Additional files 1 and 2 for the original questionnaire in Dutch and an English translation). One particular instrument that has been widely used in the Netherlands for the assessment of patient satisfaction, the Dutch Consumer Quality Index [45,46], served as the questionnaire’s framework (e.g., form of questions, response categories). The final questionnaire consisted of 62 items: 29 regarding satisfaction with the care services provided to care recipients; and 33 regarding informal caregiver support services. The majority of items were designed using a Likert scale ranging from 1 (never) to 5 (always). Some of the items could only be answered with yes/no. Other questions demanded additional response categories: ‘I do not know/No experience with that’, ‘not applicable’, and ‘not applicable/not necessary’. Items regarding age, sex and whether the informal caregiver lived with the care recipient were included as control variables [15]. In addition, the questionnaire contained several blank lines to allow subjects to note any additional comments. Filling out the questionnaire took an average of 20 minutes.

### Analysis

Analysis of the data occurred at the item level as well as at the dimension level. Before the analysis, all items containing more than 10% missing values were excluded. The Consumer Quality Index dictated that some items had to be recoded [47]. For items containing 2 response categories (no/yes), ‘no’ was recoded as 1 = never and ‘yes’ as 4 = always. Items 27, 33, 38, 45, 57 and 58 were coded in this manner. For items containing 3 response categories (never, once a year, several times a year), ‘never’ remained the same, and the 2 remaining categories were recoded as 4 = always. Items 14 and 43 were coded in this manner. Items 28 and 61 were contra-indicatively formulated. These items were re-coded in reverse order, allowing a low score to indicate low satisfaction.

#### Analysis at item level

Within-group changes in item scores between T0 and T1 were determined using paired t-tests, McNemar’s test or Wilcoxon’s signed ranked test. For between-groups comparison, independent t-tests and the Chi-square tests (or Fisher’s exact test) were used. The effect of the WICM was evaluated with linear regression or logistic analyses for each item. To assess the individual influences of variables, regression analysis was performed using 3 consecutive models. Model 1 contained the baseline measurement of the relevant item, Model 2 contained the control variables (age, sex, living together with the care recipient), and Model 3 contained the intervention as a variable. The significance of each model was assessed (<0.05). Individual effects were then assessed using a significance level of p < 0.05. However, in light of the relatively small sample, effects within the range of p < 0.10 have been reported as well.

#### Factor analysis

Using factor analysis, it was investigated whether the items of each theoretical dimension indeed constituted an underlying dimension. Primary component analysis (PCA) with oblimin rotation was used to evaluate and extract the factors of each dimension. These analyses were based on T0 scores, as there were no differences in the care delivery models at baseline. The dimensions served as a starting point for the PCA, and the items that were considered to operationalize the same dimension were therefore initially assessed for an underlying factor. Some dimensions lacked a 1-factor structure and therefore could not be made into a scale using their intended items. The subsequent process entailed the iterative inclusion and exclusion of remaining items in other scales to determine their potential fit. An important aspect of this iterative process was the alternation between quantitative and qualitative interpretation of the resulting scales. The contribution of adding and deleting items to the strength and reliability of the scales was assessed. After this quantitative assessment, the content of the items of the resulting scale was interpreted, thereby ensuring the qualitative consistency of the items. This iterative process of interpretation continued until the resulting items optimally represented their dimensions, both quantitatively and qualitatively. Items that could not be included in any scale were grouped together and assessed for underlying factors, to determine the existence of a potential new scale. Factor structures were checked by obtaining eigenvalues (>1) and scree plots. To assess the fit and significance, the KMO-Bartlett test (>0.6) and Bartlett’s test of sphericity (p < 0.05) were performed [48]. Factor loadings of >0.4 were considered sufficiently high. After the factor analysis, scales were constructed. Scales with a Cronbach’s alpha of >0.60 were considered to be reliable.

#### Analysis of scales

Scores for the resulting scales were calculated by computing the mean score for each respondent. The maximum for the missing values was one third of the items of a particular scale (half of the items for scales containing 4 items) [47]. The absence of a response constituted a missing value (not in case of ‘not applicable’). As with the analyses of the items, the outcomes per scale were analyzed with t-tests, McNemar’s test or Wilcoxon’s signed ranked test, independent t-tests and Chi-square tests (or Fisher’s exact test) and linear and logistic analyses. The regression models contained Model 1 (baseline score of scale), Model 2 (control variables) and Model 3 (the intervention).

## Results

### Response and study population

A total of 377 patients participated in the intervention with an average age of 82 years and an average GFI frailty score of 6/15. The majority of patients was female (65%), lived alone (61%) and lived independently (77%). Of these patients, 220 indicated to receive informal care. After identification, these informal caregivers were approached by mail or telephone (Table 1). The response rate of informal caregivers at T0 was relatively low at 47% (n = 104). A subsequent analysis of non-response indicated that this was primarily due to the fact that a substantial proportion of the care recipients received care from only one care professional or organization and therefore informal caregivers judged themselves unable to adequately assess (coordinated) services. An additional loss to follow-up of 39% (n = 41) between T0 and T1 resulted in a definitive study population of 63 respondents: 36 in the experimental group and 27 in the control group. This was substantially lower than expected in advance, as it was assumed that each group would contain approximately 150 patients and an equal amount of informal caregivers. Given a medium effect size of 0.15, significance of 5% and 5 independent variables, this would yield a power of 0.97. Due to the smaller sample, power was reduced to 0.60. Loss to follow-up was primarily the result of terminal illness or the death of the care recipient, the respondents not categorizing themselves as informal caregivers or changes in contact information. The majority of the definitive study population was female and did not live together with the care recipients. The respondents in the control group were significantly older than the respondents in the experimental group.

**Table 1:** Response, loss to follow-up and description of study population

	**Experimental group**	**Control group**
Informal caregivers approached	117	103
Response at T0	55	49
Loss to follow-up	19	22
Definitive study population	36	27
Age#	58 (sd = 9.5)	62 (sd = 9.5)
Male	19%	30%
Female	81%	70%
Living together (yes)	11%	15%

#p < 0.10; sd = standard deviation.

### Analysis at item level

The item regarding the rating of support for informal caregivers in general (0-10) was excluded from further analysis due to a large number of missing values. The groups showed differences on several separate items at both T0 and T1. In addition, a number of within and between-group differences were observed for both the experimental and control group. See Additional file 3 for an overview of scores on all items at T0 and T1 and the analysis of within and between-group differences.

Regression analyses for each item showed that the WICM resulted in an increase in satisfaction with the degree to which care was provided according to the wishes of the care recipient (p = 0.003) (Table 2). Conversely, the model resulted in a decrease in satisfaction with the amount of help provided with administrative tasks (p = 0.019). In addition, the model showed a decrease in satisfaction with the understandability of the information provided (p = 0.070) and the degree to which informal caregivers knew which professional to call in cases of complaints, problems or emergencies (p = 0.091). For all of the items, T0 scores were the main predictor of scores at T1. In addition, the results showed that female informal caregivers were less satisfied with the degree to which care was provided according to the wishes of care recipients (p = 0.049), and older informal caregivers knew better which professionals to call (p = 0.087). See Additional files 4 and 5 for an overview of regression analyses on all items.

**Table 2:** Adjusted R2, β and p-values of dependent variables in regression analysis of item scores

**Satisfaction with care**	**Adj. R2**	**T0**	**Age**	**Sex**	**LT**	**WICM**
Care provided according to wishes	30%	.37**	.14	-.29*	-.12	.38**
Sufficient help with administrative tasks	37%	.58**	-.25	.07	.27	-.45*
**Satisfaction with support**	**Adj. R2**	**T0**	**Age**	**Sex**	**LT**	**WICM**
Understand information	23%	.44**	-.10	-.03	.20	**-.24#**
Know who to call	29%	.36**	**.27#**	-.20	.02	**-.20#**

#p < 0.10; *p < 0.05, **p < 0.01; ***p < 0.001 (shown in bold).

LT = living together; WICM = Walcheren Integrated Care Model.

### Principal component analysis

The principal component analysis indicated that the resulting scales only partly overlapped with the dimensions derived from literature. The Kaiser-Meyer-Olkin measurement verified the sampling adequacy, which ranged from 0.66 to 0.86. Bartlett’s test of sphericity indicated that the correlations between the items of each scale were sufficiently strong (p < 0.05).

Satisfaction with care resulted in 6 scales (Table 3). Three items could not be included in any scale: satisfaction with the frequency with which professionals visited the care recipient; satisfaction with the promptness of services; and a general rating of the care provided. Items regarding satisfaction with the support provided to informal caregivers were categorized into 5 scales (Table 4). Five items could not be included in any scale: understanding the information provided; having control over one’s role and care tasks; the evaluation of care by professionals with the informal caregiver; information provision regarding in-home adaptations; and assistance in finding activities.

**Table 3:** Factor loadings of items on the scales for satisfaction of informal caregivers with the care provided to care recipients

**Scales**						
**Items**	**Care arrangements**	**Information/involvement**	**Personal interaction**	**Professionalism**	**Client-centeredness**	**Sufficient assistance**
Professionals use care plan	.843					
Professionals coordinate visits	.739					
Professionals adhere to arrangements	.693					
Time of visits is suitable	.653					
Professionals involve care recipient in decisions		.829				
Professionals provide sufficient information		.804				
Care recipient understands information		.683				
Professionals evaluate care process frequently		.590				
Professionals coordinate care tasks			.767			
Professionals respond adequately to questions			.707			
Professionals are attentive to needs			.691			
Professionals are polite			.684			
Professionals have sufficient time			.612			
Professionals know needs of care recipient				.809		
Professionals are careful with belongings				.791		
Professionals collaborate well with others				.766		
Professionals provide good quality of care				.758		
Professionals are attentive to changes in health					.861	
Professionals are attentive to overall well-being					.833	
Professionals take functional ability into account					.794	
Professionals provide care according to wishes					.597	
Professionals help sufficiently with administrative affairs						.909
Professionals pay sufficient attention to safety						.849
Professionals help sufficiently with finding activities						.798
Professionals provide sufficient (emotional) support						.772
The care recipient receives sufficient care						.691
KMO	.666	.711	.768	.721	.716	.812
Bartlett’s Chi-square test	27.664***	23.591**	34.417***	51.327***	61.764***	42.232***
Eigenvalue	2.164	2.147	2.410	2.441	2.423	3.259
% variance	54%	54%	48%	61%	61%	65%
**α** T0	.71	.68	.71	.78	.78	.85
**α** T1	.73	.77	.80	.86	.78	.75

**p < 0.005; ***p < 0.000.

**Table 4:** Factor loadings of items on the scales for satisfaction of informal caregivers with their support

**Scales**					
**Items**	**Information**	**Being involved**	**Professionalism**	**Communication/accessibility**	**Attention to health/support**
Professionals provide sufficient information: care	.938				
Professionals provide sufficient information: services	.931				
Professionals provide sufficient information: expectations	.901				
Professionals provide sufficient information: how to care	.866				
Arrangements for emergency situations	.722				
Professionals discusses tasks with caregiver		.933			
Sufficiently involved in care decisions		.883			
Availability of professionals in case of problems		.853			
Professionals make arrangements if care changes		.845			
Professionals react adequately to questions/suggestions			.870		
Feel safe in proximity of professionals			.867		
Professionals take me seriously			.865		
Professionals have sufficient time			.863		
Professionals listen carefully			.863		
Professionals are attentive to needs			.851		
Professionals are polite			.613		
Professionals are easily accessible by phone				.964	
One professional/contact point				.875	
Know where to go/whom to go to with complaints				.861	
Information is provided about waiting time				.837	
Professionals keep each other informed				.631	
Professionals take needs into account					.974
Sufficient assistance and support are provided					.937
Professionals pay attention to changes in health					.930
Professionals pay sufficient attention to well-being					.922
Professionals provide sufficient emotional support					.852
Professionals take functional abilities into account					.850
KMO	.856	.827	.831	.762	.813
Bartlett’s Chi-square test	98.932***	91.356***	193.426***	37.108***	91.119***
Eigenvalue	3.830	3.091	4.846	3.535	4.988
% variance	77%	77%	69%	71%	83%
**α** T0	.92	.89	.92	.86	.96
**α** T1	.86	.83	.82	.62	.92

**p < 0.005; ***p < 0.000.

### Analysis at scale level

#### Within-group and between-groups analysis

No significant changes were found for scores on the scales for informal caregiver satisfaction with the care provided to the care recipient (Table 5), which was also the case for the scores for T1, for the difference between T0 and T1 within the groups and for changes between the groups. However, differences were found for the scales rating satisfaction with support. Within-groups analysis revealed that scores on the scale of ‘being involved’ significantly increased between T0 and T1 for both the experimental (p = 0.048) and control (p = 0.086) groups. Scores on the scale of ‘attention to health and support’ were higher at T1 for the experimental group (p = 0.058). However, these changes over time did not result in any significant differences between the experimental and the control group.

**Table 5:** Within and between-group analysis of scale mean scores at T0 and T1

**Satisfaction with care**	**Experimental group**			**Control group**			**Δ Group**
	**T0 (sd)**	**T1 (sd)**	**Δ**	**T0 (sd)**	**T1 (sd)**	**Δ**	***p***
Care arrangements	3.3 (0.48)	3.3 (0.52)	0.03	3.4 (0.47)	3.3 (0.39)	-0.09	-
Information/involvement	3.3 (0.60)	3.2 (0.73)	-0.12	3.2 (0.69)	3.3 (0.41)	0.07	-
Personal interaction	3.3 (0.46)	3.4 (0.46)	0.07	3.4 (0.31)	3.4 (0.38)	0.02	-
Professionalism	3.5 (0.50)	3.4 (0.57)	-0.06	3.6 (0.45)	3.5 (0.43)	-0.05	-
Client-centeredness	3.4 (0.55)	3.4 (0.54)	0.05	3.5 (0.46)	3.4 (0.36)	-0.10	-
Additional assistance	2.9 (0.76)	2.9 (0.72)	-0.04	2.8 (0.85)	3.0 (0.55)	0.23	-
**Satisfaction with support**							
Information	2.1 (1.08)	1.9 (0.98)	-0.18	2.6 (1.30)	2.2 (0.88)	-0.41	-
Being involved	2.5 (0.92)	2.8 (0.87)	0.31*	2.8 (0.93)	3.1 (0.72)	0.25#	-
Professionalism	3.3 (0.54)	3.4 (0.39)	0.15	3.4 (0.44)	3.4 (0.45)	0.00	-
Communication/accessibility	3.2 (0.81)	3.2 (0.88)	-0.01	3.3 (0.78)	3.5 (0.58)	0.12	-
Attention to health/support	2.2 (1.08)	2.7 (1.26)	0.50#	3.1 (0.77)	2.8 (0.82)	-0.32	-

#p < 0.10; *p < 0.05; **p < 0.01; ***p < 0.001.

sd = standard deviation; Δ = difference between T0 and T1; Δ Group = difference between groups.

#### Regression analysis for scales

The WICM did not affect any scale measuring satisfaction with care (Table 6). Satisfaction with care at T1 was primarily a function of satisfaction with care at T0, with baseline scores of the scales showing significance values ranging from 0.05 to 0.000. The greater the satisfaction with care was at baseline, the greater it was at follow-up. In addition, older informal caregivers were more satisfied with client-centeredness (p = 0.060). Regarding satisfaction with support, regression analyses revealed that the WICM had a marginal effect on the scale of ‘attention to health and support’, with a p-value just greater than the significant range (p = 0.10). Again, baseline scores were the main predictor of the scales and items measuring satisfaction with support, with significance values ranging from 0.01 to 0.000. An additional positive effect was found for living together on satisfaction with the degree of involvement (p = 0.051). For the scale of ‘professionalism (IC)’, the regression model was not significant.

**Table 6:** **Adjusted R2, β and *p*-values for all scales**

**Scales CR**	**Adj. R2**	**T0**	**Age**	**Sex**	**LT**	**WICM**
Care arrangements	26%	.43**	-.26	-.08	-.03	-.06
Information/involvement	30%	.59**	-.04	-.01	-.19	-.12
Communication	18%	.39*	.12	-.17	-.00	.09
Professionalism (CR)	32%	.51***	.21	-.11	-.07	.02
Client-centeredness	43%	.57***	**.27#**	-.17	-.07	.17
Additional assistance	43%	.48***	.27	-.11	-.02	-.12
**Scales IC**	**Adj. R2**	**T0**	**Age**	**Sex**	**LT**	**WICM**
Information	55%	.57***	-.06	-.22	.27	-.09
Involvement	47%	.53***	.14	.09	**.27#**	-.03
Professionalism (IC)	n.s.	**-**	-	-	-	-
Communication/accessibility	30%	.48***	.14	-.09	.09	-.11
Attention to health/support	61%	.73**	.29	-.04	.14	.35

#p < 0.10; *p < 0.05: **p < 0.01; ***p = 0.000.

LT = live together; WICM = Walcheren Integrated Care Model.

CR = care recipient; IC = informal caregiver; n.s = model not significant.

## Discussion

This study explored the effects of the Walcheren Integrated Care Model on the satisfaction of informal caregivers with the care provided to elderly care recipients and with the support the caregivers received themselves. The WICM had no substantial effect on informal caregiver satisfaction with care and support services. At the item level, an increase was observed in the satisfaction with the degree to which care was provided according to the needs of care recipients. In addition, the WICM resulted in decreased satisfaction with the degree to which professionals provided sufficient help with administrative tasks, the understandability of the information provided and the degree to which informal caregivers knew which professional to call in cases of problems, complaints or emergencies.

The positive effects that were found suggest that from the informal caregiver’s perspective, integrated care has the potential to provide care according to the needs of the care recipient. This finding provides some confirmation of one of the major objectives of integrated care [19]. The negative results were rather surprising, as the WICM explicitly aimed to address issues of transparency and information provision. In addition, the observed adverse effects were not in agreement with other studies, which reported increased caregiver satisfaction as a result of integrated care interventions similar to the WICM [15,18]. Both studies reported on the same intervention (SIPA), which included patient screening, care plan development, case management, a multidisciplinary team, protocols and a single entry point [49]. However, the SIPA intervention did not include the explicit involvement of informal caregivers in the planning and provision of care, while the WICM did. Moreover, the WICM paid substantial attention to the optimization of information provision to informal caregivers regarding available services and how to obtain these services. This difference between the WICM and SIPA intervention might also provide some explanation for the negative effects observed in the current study. Perhaps the additional efforts with the WICM to maximize information provision to informal caregivers regarding available services and how to obtain these services, as well as advice regarding how to perform certain care tasks adequately, were experienced by informal caregivers as interference. Potentially, the sum of such well-intentioned efforts might have actually been counterproductive, resulting in information overload, thus reducing the understandability of the information provided and increasing the uncertainty and confusion of informal caregivers. Such counterproductive effects were described by Winslow [50], who noted that information overload by formal services is often experienced by informal caregivers as a major ‘hassle’. Similarly, it is conceivable that the introduction of a case manager reduced clarity for informal caregivers regarding which professional would be the most appropriate to call in cases of problems, complaints and emergencies. While case management in the WICM aimed to provide a central source of information, one-on-one professional guidance and ongoing case coordination, such counterproductive mechanisms could not be ruled out. Indeed, Fabbricotti [51] noted that the introduction of coordinating roles, such as a case manager, could actually decrease clarity for care recipients and their informal caregivers, as they would need to interact with yet another professional.

Another explanation for our results might be provided by our instrument. While the SIPA studies [15,18] used a measurement of general satisfaction [41], the current study constructed an instrument that was thought to be more sensitive to various service elements, specifically those associated with integrated care. In addition, our approach incurred the risk of assessing satisfaction on a range that might have been too broad. Perhaps our instrument contained items regarding services that respondents simply had no experience with, in which case it would have been difficult to find effects. Another study regarding the effects of integrated care on informal caregiver satisfaction also used a self-constructed instrument [24]. However, those authors did not provide a description of the process of questionnaire construction or of the content of the questionnaire, making interpretation of their results difficult. Another issue related to the measurement of satisfaction is the fact that studies of satisfaction tend to produce high scores, making it difficult to detect changes (e.g., [15]). In addition, satisfaction scores are often robust over time, evidenced in the current study by T0 scores being the best predictor of T1 scores. However, as the range of scores in this study provided sufficient room for variance, any occurrence of improvements would have been detected.

A final explanation might be provided by the unequal distribution of co-residing and non-co-residing informal caregivers in the study population. The majority of our population did not live with care recipients, perhaps reducing the likelihood of interaction occurring between caregivers and formal services and professionals. In such cases, informal caregivers would have lacked experience with important characteristics of services, such as client-centeredness, professionalism and the manner and content of communication. Without such experiences, informal caregivers would not have been able to adequately assess these services, making it difficult to validly assess their satisfaction. In addition, it has been noted that spousal informal caregivers, i.e., those who co-reside with the care recipient, assess services differently than those who do not co-reside [4]. However, whether this difference affected our results remains unclear, as the relatively small sample of this study did not allow for subgroup analyses [52].

Some secondary results were observed, such as a reduction in satisfaction with the degree to which the care recipient’s needs were taken into account for female informal caregivers and better knowledge of which professional to call in cases of emergency, problems or complaints for older informal caregivers. Co-residing informal caregivers showed greater satisfaction on the scale of being involved by professionals, and older informal caregivers showed greater satisfaction on the scale of client-centeredness.

### Limitations

Constructing an instrument for a specific study context inherently entails a trade-off with the validity of the instrument, which constituted a limitation of the current study. However, while the benefits of validated instruments, such as the SCQ-8 [41], are evident, its use would require adaptations for informal caregivers (e.g., [15,18,42]), thereby substantially undermining the instrument’s validity. The relatively small variance that was explained by the regression models indicated the existence of other control variables. Indeed, other variables have been shown to be associated with informal caregiver satisfaction with care services, such as increased level of impairment or more frequent disruptive behavior of the care recipient, the informal caregiver being part of a cultural minority in a country [53], education, marital status, social status [15] and employment status [54]. The relatively small sample size was another limitation as it substantially reduced the statistical power. The observed trend in increased satisfaction with attention to needs and health suggests that an effect might have been found with a larger sample [55]. Differences between groups may constitute a final limitation. Besides the observed age difference, it seemed that there was some overall difference in satisfaction at baseline. This makes it more challenging to show effects of the intervention.

### Are the expectations justified?

This study raises the question of whether the existing expectations of the effects of integrated care on informal caregiver satisfaction are justified. Specifically, the lack of substantial positive effects, in addition to some negative effects, found in this study suggests that the assumption that integrated care increases informal caregiver satisfaction might not necessarily be true. While there is some evidence for positive effects, studies have simply been too scarce to draw any decisive conclusions. Moreover, this scarcity of evidence is in stark contrast with the substantial body of literature regarding integrated care. In the absence of evidence, the possibly inflated expectation of the beneficial effects of integrated care on informal caregiver satisfaction will continue to exist without being contested. In this sense, the debate on integrated care and informal caregiver satisfaction could benefit if the academic community would be more attentive to adverse effects. To this end, studies yielding no or negative results should be equally eligible for publication as those yielding positive results. As both integrated care and informal care have become major priorities in research and policy agendas, this need is even more urgent. Researchers and policymakers might need to consider the possibility that under some conditions, including informal caregivers in integrated care arrangements may have a downside. In other words, we should not readily assume that more informal caregiver involvement is always better, as the opposite might be true: perhaps less is more.

### Recommendations

We recommend the development and validation of a comprehensive instrument to assess informal caregiver satisfaction with services. The resulting scales in the current study might provide guidance in this process. Future studies should also consider including a broader range of control variables. We also propose that in the design and implementation of integrated care arrangements, the possibility of adverse effects on informal caregiver satisfaction is considered. In addition, future studies of integrated care should investigate the assumption that co-residing informal caregivers react differently to interventions than caregivers who do not co-reside. This goal might be achieved by including a study population that is sufficiently large to allow for adequate subgroup analyses. Furthermore, it is recommended that the issue of co-residence also be taken into account in future integrated care interventions. This goal could be met by mapping the specific needs of co-residing informal caregivers qualitatively prior to designing the intervention.

## Conclusion

The WICM did not substantially affect informal caregivers’ satisfaction with the care for the care recipient or their satisfaction with the support the caregivers received themselves. The question can be raised whether the expectations regarding the beneficial effect of integrated care on informal caregiver satisfaction are justified.

## Abbreviations

WICM: Walcheren Integrated Care Model; GP: General practitioner; PCP: Primary care practice; CSQ: Client Satisfaction Questionnaire; PCA: Primary component analysis; SIPA: Système de services intégrés pour personnes âgées en perte d’antonomie.

## Competing interests

The authors declare that they have no competing interests.

## Authors’ contributions

BJ collected the data, performed the statistical analyses and wrote the paper. As the project leader of the study, IF planned and designed the study, supervised the data collection, performed statistical analyses and contributed to revising the paper. RH contributed to initiating the research project and revising the paper. All of the authors read and approved the final manuscript.

## Pre-publication history

The pre-publication history for this paper can be accessed here:

http://www.biomedcentral.com/1472-6963/14/140/prepub

## Supplementary Material

Additional file 1 The questionnaire as developed for the current study (English translation). Click here for file

Additional file 2 The questionnaire as developed for the current study (in Dutch). Click here for file

Additional file 3 **Within- and between-group analysis of mean item scores at T0 and T1.** Description of file: Table showing mean scores, standard deviations and significant changes within and between the experimental and control groups. Click here for file

Additional file 4 **Adjusted R2, β and ***p***-values for items regarding satisfaction with care provided to care recipients (CR).** Description of file: Results of regression analyses on items regarding care to the care recipient. Click here for file

Additional file 5 **Adjusted R2, β and *p*-values for items regarding satisfaction with support of informal caregivers (IC).** Description of file: Results of regression analyses on items regarding care to the informal caregiver. Click here for file
