# Supplementary material for: A quasi-experimental study of the effects of an integrated care intervention for the frail elderly on informal caregivers’ satisfaction with care and support
Source: BMC Health Serv Res. 2014 Mar 29;14:140. doi: 10.1186/1472-6963-14-140 (PMC3986650; doi:10.1186/1472-6963-14-140)
Supplement: Additional file 1 — The questionnaire as developed for the current study (English translation). [file 1472-6963-14-140-S1.doc]

[
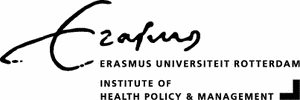
](http://www.bmg.eur.nl/fileadmin/ASSETS/bmg/intranet/marketing_communicatie_pr/huisstijl/iBMG_logo_ZW_UK.png)

# **Informal Caregiver Satisfaction**

# **Questionnaire**

# **Walcheren Integrated Care Model**

**Instructions**

This questionnaire consists of 2 parts:

- The first part concerns your satisfaction with the care received by your loved one. **What is your opinion about the care he/she receives?**
- The second part concerns your satisfaction with the care and support you receive yourself. **What is your opinion about the care and support that you receive?**
- Filling out this questionnaire will take about 20 minutes
- ‘Your loved one’ is the person that you care for as informal caregiver
- Please tick the box of the answer that most applies to you

**What is your opinion about the care that your loved one receives?**

###### Care arrangements

1. **Do professionals adhere to the arrangements that have been made?**

- never
- sometimes
- mostly
- always
- not applicable / I don’t know

1. **Do professionals use the care plan to exchange information? (e.g. look what other professionals have written, call other professionals in relation to what has been written in care plan)**

- never
- sometimes
- mostly
- always
- not applicable/ I don’t know

1. **Do professionals coordinate with each other who and when visits your loved one (day, time)?**

- never
- sometimes
- mostly
- always
- not applicable/ I don’t know

1. **Does the time and day on which care is provided suit your loved one?**

- never
- sometimes
- mostly
- always
- not applicable/ I don’t know

1. **Do professionals involve your loved one in decisions regarding care?**

- never
- sometimes
- mostly
- always
- not applicable/ I don’t know

###### Information

1. **Do professionals provide sufficient information about care? (e.g. options for home care, available services)**

- never
- sometimes
- mostly
- always
- not applicable/ I don’t know

1. **Does your loved one understand the information about care that is provided by professionals?**

- never
- sometimes
- mostly
- always
- not applicable/ I don’t know

###### Communication

1. **Are the professionals sufficiently open to the wishes of your loved one?**

- never
- sometimes
- mostly
- always
- not applicable/ I don’t know

1. **In your opinion, do professionals react adequately to the questions of your loved one?**

- never
- sometimes
- mostly
- always
- not applicable/ I don’t know

1. **Are professionals polite to your loved one?**

- never
- sometimes
- mostly
- always
- not applicable/ I don’t know

1. **Do professionals have enough time for your loved one?**

- never
- sometimes
- mostly
- always
- not applicable/ I don’t know

###### Coordination and substitution

1. **What is your opinion of the amount of professionals visiting your loved one?**

- too few
- precisely enough
- a lot, but acceptable
- too much
- not applicable / I don’t know

1. **Do professionals coordinate care adequately?**

- never
- sometimes
- mostly
- always
- not applicable/ I don’t know

###### Evaluation of care

1. **Do professionals evaluate care with your loved one? (e.g. sufficient care, satisfaction)**

❑ never

❑ once a year

❑ several times a year

- not applicable / I don’t know

###### Professionalism

1. **Are professionals competent? (do they provide quality care)**

- never
- sometimes
- mostly
- always
- not applicable/ I don’t know

1. **Are professionals adequately informed about the disabilities and health problems of your loved one?**

- never
- sometimes
- mostly
- always
- not applicable/ I don’t know

1. **Do professionals collaborate adequately with each other? (e.g. general practitioner, physiotherapist, dietician)**

- never
- sometimes
- mostly
- always
- not applicable/ I don’t know

1. **Do professionals handle the possessions of your loved one carefully? (e.g. furniture, tableware , clothing)**

- never
- sometimes
- mostly
- always
- not applicable/ I don’t know

###### Care and health

1. **Do professionals provide care according to the wishes of your loved one?**

- never
- sometimes
- mostly
- always
- not applicable/ I don’t know

1. **Do professionals take the functional abilities of your loved one into account? (what he/she can and cannot do)**

- never
- sometimes
- mostly
- always
- not applicable/ I don’t know

1. **Are professionals attentive to changes in the health of your loved one?**

- never
- sometimes
- mostly
- always
- not applicable/ I don’t know

1. **Do professionals pay sufficient attention to the general well-being of your loved one?**

- never
- sometimes
- mostly
- always
- not applicable/ I don’t know

1. **Do professionals provide sufficient emotional support to your loved one?**

- never
- sometimes
- mostly
- always
- not applicable/ I don’t know

###### Safety

1. **Do professionals pay sufficient attention to the safety of your loved one? ( e.g. prevention of accidents, attention to expiration dates of food)**

- never
- sometimes
- mostly
- always
- not applicable/ I don’t know

###### Independency and activities

1. **Do professionals provide sufficient help in finding activities and services?**

- never
- sometimes
- mostly
- always
- not applicable/ I don’t know

1. **Do professionals provide sufficient assistance with administrative tasks? (e.g. tax forms, applications for in-home devices, bookkeeping)**

- never
- sometimes
- mostly
- always
- not applicable/ I don’t know

1. **In your opinion, is the amount of care received by your loved one sufficient?**

❑ yes

❑ no

###### Waiting time

1. **In general, the waiting time in the care provision to your loved one is:**

❑ short

❑ acceptable

❑ somewhat long

- too long
- not applicable: my loved one always receives care instantly

###### General rating of professionals

1. **What grade would you give the professionals that provide care to your loved one? 0= very bad, 10= excellent.**

- 0 *very bad*
- 1
- 2
- 3
- 4
- 5
- 6
- 7
- 8
- 9
- 10 *excellent*

The following questions concern your experience with the care and support that professionals provide to you. ***What is your opinion about the care and support that you receive?***

###### Care arrangements and information

1. **Do professionals sufficiently involve you in decisions regarding care to your loved one? (e.g. nature of tasks or activities, time of visits)**

- never
- sometimes
- mostly
- always
- not applicable/ I don’t know

1. **Do professionals provide you with sufficient information regarding the care to your loved one? ( e.g. options for home-care, available services)**

- never
- sometimes
- mostly
- always
- not applicable/ I don’t know

1. **Do professionals provide you with sufficient information regarding where to go for care or assistance if they cannot provide it themselves? (e.g. provide phone number of social care services)**

- never
- sometimes
- mostly
- always
- not applicable/ I don’t know

1. **Do professionals provide you with enough information regarding what is expected of you as an informal caregiver? (e.g. what to arrange, financial contributions)**

❑ no

❑ yes

1. **Do professionals provide you with sufficient information regarding how to provide care to your loved one?**

- never
- sometimes
- mostly
- always
- not applicable/ I don’t know

1. **Do you understand the information that is provided by the professionals?**

- never
- sometimes
- mostly
- always
- not applicable/ I don’t know

1. **Do you have control over your role and your tasks in the care to the your loved one?**

- never
- sometimes
- mostly
- always
- not applicable/ I don’t know

###### Communication

1. **Do you know who to contact if you have questions, problems or complaints?**

- never
- sometimes
- mostly
- always
- not applicable/ I don’t know

1. **Do you have a central and fixed contact person/point you can go to with your questions?**

❑ yes

❑ no

1. **Are professionals sufficiently open to your wishes?**

- never
- sometimes
- mostly
- always
- not applicable/ I don’t know

1. **In your opinion, do professionals react adequately to your questions and suggestions?**

- never
- sometimes
- mostly
- always
- not applicable/ I don’t know

1. **Do professionals discuss with you what tasks need to be done?**

- never
- sometimes
- mostly
- always
- not applicable/ I don’t know

1. **Are professionals generally easy to reach by telephone?**

- never
- sometimes
- mostly
- always
- not applicable/ I don’t know

###### Evaluation of care

1. **Do professionals evaluate your satisfaction with the care to your loved one?**

- never
- once a year
- several times a year
- not applicable/ I don’t know

1. **Do professionals make new arrangements with you when your love one needs less or more care?**

- never
- sometimes
- mostly
- always
- not applicable/ I don’t know

1. **Do professionals keep each other informed about changes in the care they provide to your loved one, or do you often need to tell such things to them?**

- professionals contact each other regarding changes in care
- I often notice that they are uninformed regarding such changes, so I tell
- not applicable/ I don’t know

1. **Do you have sufficient opportunity to turn to professionals in case you run into any problems?**

- never
- sometimes
- mostly
- always
- not applicable/ I don’t know

###### Professionalism

1. **Are professionals polite to you?**

- never
- sometimes
- mostly
- always
- not applicable/ I don’t know

1. **Do professionals have enough time for you?**

- never
- sometimes
- mostly
- always
- not applicable/ I don’t know

1. **Do professionals listen attentively to you?**

- never
- sometimes
- mostly
- always
- not applicable/ I don’t know

1. **Do professionals take you seriously?**

- never
- sometimes
- mostly
- always
- not applicable/ I don’t know

###### Care and health

1. **Do you receive sufficient support and assistance from professionals?**

- never
- sometimes
- mostly
- always
- not applicable/ I don’t know

1. **Do professionals sufficiently take your functional abilities into account?**

- never
- sometimes
- mostly
- always
- not applicable/ I don’t know

1. **Do professionals pay sufficient attention to changes in your health?**

- never
- sometimes
- mostly
- always
- not applicable/ I don’t know

1. **Do professionals pay sufficient attention to your general well-being?**

- never
- sometimes
- mostly
- always
- not applicable/ I don’t know

1. **Do professionals provide sufficient emotional support to you?**

- never
- sometimes
- mostly
- always
- not applicable/ I don’t know

1. **Do professionals sufficiently take your needs into account?**

- never
- sometimes
- mostly
- always
- not applicable/ I don’t know

###### Safety

1. **Do professionals provide you with information regarding in-home adjustments and other devices?**

❑ no

❑ yes

- not applicable/ not necessary

1. **Did professionals discuss with you what to do in case of emergency? (e.g. phone number, how to use alarm system)**

❑ no

❑ yes

- not applicable/ not necessary

1. **Do you feel safe and comfortable in the presence of professionals?**

- never
- sometimes
- mostly
- always
- not applicable/ not necessary

1. **Do professionals provide sufficient help in finding services for both your loved one and yourself? (e.g. day care, activities, group counseling)**

- never
- sometimes
- mostly
- always
- not applicable/ not necessary

###### Waiting time

1. **Do professionals provide you with sufficient information regarding waiting times for certain care services and what you can do in the meantime?**

- more than sufficient
- sufficient
- insufficient
- completely insufficient
- not applicable (no waiting time)

###### General rating of the care and support you have received

1. **What grade would you give the professionals for the care and support they provided to you? 0= very bad, 10=excellent.**

- 0 *very bad*
- 1
- 2
- 3
- 4
- 5
- 6
- 7
- 8
- 9
- 10 *excellent*

1. **What changes would you like to see in the care provision to your loved one?**

**67. What changes would you like to see in the care and support that is provided to you?**

**Thank you very much for your cooperation!**
