# Supplementary material for: A quasi-experimental study of the effects of an integrated care intervention for the frail elderly on informal caregivers’ satisfaction with care and support
Source: BMC Health Serv Res. 2014 Mar 29;14:140. doi: 10.1186/1472-6963-14-140 (PMC3986650; doi:10.1186/1472-6963-14-140)
Supplement: Additional file 2 — The questionnaire as developed for the current study (in Dutch). [file 1472-6963-14-140-S2.doc]

# **
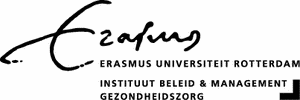
**

**CODE**

# **Tevredenheid mantelzorger**

# **project Ketenzorg Ouderen Walcheren**

**iBMG**

**Aanwijzingen bij deze vragenlijst**

De vragenlijst bestaat uit twee onderdelen:

- In het eerste deel vragen wij u naar uw tevredenheid met de zorg voor uw naaste. ***Wat vindt u van de zorg die uw naaste krijgt?***
- In het tweede deel vragen wij u hoe u de zorg en de begeleiding door de zorgverleners van uw naaste aan uzelf ervaart. ***Wat vindt u van de zorg en begeleiding die u zelf krijgt?***
- Het invullen van de vragenlijst kost u ongeveer 20 minuten
- Als u leest 'uw naaste' dan gaat de vraag over de persoon voor wie u mantelzorger bent
- U kunt het antwoord aankruisen dat het beste bij u past

De volgende vragen gaan over hoe u de zorg door zorgverleners aan uw naaste ervaart. ***Wat vindt u van de zorg die uw naaste krijgt?***

###### Afspraken over zorg

1. **Komen de zorgverleners de afspraken over de zorg goed na?**

- nooit
- soms
- meestal
- altijd
- niet van toepassing/weet ik niet

1. **Gebruiken de zorgverleners het behandelplan of zorgplan goed voor het uitwisselen van informatie? (kijken zij wat een ander erin heeft geschreven, bellen ze iemand op op basis van wat zij lezen, enz)**

- nooit
- soms
- meestal
- altijd
- niet van toepassing/weet ik niet

1. **Stemmen zorgverleners onderling af wie op welke dag of welk tijdstip komt?**

- nooit
- soms
- meestal
- altijd
- niet van toepassing/weet ik niet

1. **Schikken de tijdstippen en dagen waarop uw naaste de zorg krijgt?**

❑ nooit

❑ soms

❑ meestal

- altijd
- niet van toepassing/weet ik niet

1. **Wordt uw naaste betrokken bij beslissingen over de zorg?**

❑ nooit

❑ soms

❑ meestal

- altijd
- niet van toepassing/weet ik niet

###### Informatie

1. **Geven de zorgverleners uw naaste voldoende informatie over de zorg? (de mogelijkheden voor zorg thuis, het dienstenpakket, enz.)**

❑ nooit

❑ soms

❑ meestal

- altijd
- niet van toepassing/weet ik niet

1. **Begrijpt uw naaste de informatie die zorgverleners geven over de zorg?**

❑ nooit

❑ soms

❑ meestal

- altijd
- niet van toepassing/weet ik niet

###### Communicatie

1. **Staan de zorgverleners voldoende open voor de wensen van uw naaste?**

❑ nooit

❑ soms

❑ meestal

❑ altijd

- niet van toepassing/weet ik niet

1. **Vindt u dat de zorgverleners goed reageren op vragen van uw naaste?**

❑ nooit

❑ soms

❑ meestal

❑ altijd

- niet van toepassing/weet ik niet

1. **Zijn de zorgverleners beleefd tegen uw naaste?**

❑ nooit

❑ soms

- meestal
- altijd
- niet van toepassing/weet ik niet

1. **Hebben de zorgverleners genoeg tijd voor uw naaste?**

❑ nooit

❑ soms

- meestal
- altijd
- niet van toepassing/weet ik niet

###### Afstemming en vervanging

1. **Wat vindt u van het aantal verschillende zorgverleners dat bij uw naaste langskomt?**

- te weinig
- precies goed
- veel, maar acceptabel
- te veel
- niet van toepassing/weet ik niet

1. **Stemmen de verschillende zorgverleners de zorg goed op elkaar af?**

❑ nooit

❑ soms

❑ meestal

❑ altijd

- niet van toepassing/weet ik niet

###### Evaluatie zorg

1. **Bespreken de zorgverleners met uw naaste hoe de zorg bevalt? (of uw naaste tevreden is, of uw naaste genoeg zorg krijgt, enz.)**

❑ nooit

❑ 1 keer per jaar

❑ meerdere keren per jaar

- niet van toepassing/weet ik niet

###### Professionaliteit

1. **Werken de zorgverleners vakkundig? (geven zij goed zorg)**

❑ nooit

❑ soms

❑ meestal

- altijd
- niet van toepassing/weet ik niet

1. **Zijn de zorgverleners goed op de hoogte van de ziekte(n) of gezondheidsproblemen van uw naaste?**

❑ nooit

❑ soms

❑ meestal

❑ altijd

- niet van toepassing/weet ik niet

1. **Werken de zorgverleners goed samen met andere hulpverleners? Denk hierbij bijvoorbeeld aan de huisarts, een specialist, fysiotherapeut, of diëtiste.**

❑ nooit

❑ soms

❑ meestal

❑ altijd

- niet van toepassing/weet ik niet

1. **Gaan de zorgverleners zorgvuldig om met de spullen van uw naaste? (meubels, servies, kleding, enz.)**

❑ nooit

❑ soms

❑ meestal

❑ altijd

- niet van toepassing/weet ik niet

###### Verzorging en gezondheid

1. **Krijgt uw naaste de zorg op de manier zoals hij of zij wil?**

❑ nooit

❑ soms

❑ meestal

❑ altijd

- niet van toepassing/weet ik niet

1. **Houden de zorgverleners voldoende rekening met wat uw naaste wel en niet kan?**

❑ nooit

❑ soms

❑ meestal

❑ altijd

- niet van toepassing/weet ik niet

1. **Letten zorgverleners op veranderingen in de gezondheid van uw naaste?**

❑ nooit

❑ soms

❑ meestal

❑ altijd

- niet van toepassing/weet ik niet

1. **Hebben zorgverleners voldoende aandacht voor hoe het met uw naaste gaat?**

❑ nooit

❑ soms

❑ meestal

❑ altijd

- niet van toepassing/weet ik niet

1. **Bieden de zorgverleners uw naaste voldoende emotionele ondersteuning als gesprekspartner of luisterend oor?**

❑ nooit

❑ soms

❑ meestal

❑ altijd

- niet van toepassing/weet ik niet

###### Veiligheid

1. **Hebben de zorgverleners voldoende aandacht voor de veiligheid van uw naaste? (bijvoorbeeld: voorkomen van ongelukken in en om het huis, letten op houdbaarheid eten en drinken)**

❑ nooit

❑ soms

❑ meestal

❑ altijd

- niet van toepassing/weet ik niet

###### Zelfstandigheid en activiteiten

1. **Helpen de zorgverleners uw naaste voldoende bij het vinden van mogelijkheden voor dagbesteding, sociale contacten en activiteiten?**

❑ nooit

❑ soms

❑ meestal

❑ altijd

- niet van toepassing/weet ik niet

1. **Helpen de zorgverleners uw naaste voldoende bij regelzaken? (telefoneren, formulieren invullen, regelen van hulp(middelen) of financiële zaken, enz.)**

❑ nooit

❑ soms

❑ meestal

❑ altijd

- niet van toepassing/weet ik niet

1. **Vindt u dat uw naaste genoeg zorg krijgt?**

❑ ja

❑ nee

###### Wachttijd

1. **Vindt u de wachttijd voor zorg aan uw naaste gemiddeld genomen:**

❑ kort

❑ acceptabel

❑ iets te lang

- veel te lang
- niet van toepassing: mijn naaste heeft altijd direct zorg gekregen

###### Algemene beoordeling zorg aan uw naaste

1. **Welk cijfer geeft u de zorgverleners? Een 0 betekent: heel erg slecht. Een 10 betekent: uitstekend.**

- 0 *heel erg slechte zorgverleners*
- 1
- 2
- 3
- 4
- 5
- 6
- 7
- 8
- 9
- 10 *uitstekende zorgverleners*

De volgende vragen gaan over uzelf en hoe u de zorg en de begeleiding door de zorgverleners van uw naaste aan uzelf ervaart. ***Wat vindt u van de zorg en begeleiding die u zelf krijgt?***

###### Afspraken over zorg en informatie

1. **Wordt u voldoende betrokken bij beslissingen over de zorg aan uw naaste? (de soort taken, activiteiten en werkzaamheden, het tijdstip en wie de zorgverlener is)**

- nooit
- soms
- meestal
- altijd
- niet van toepassing/niet nodig

1. **Geven de zorgverleners u voldoende informatie over de zorg aan uw naaste? (de mogelijkheden voor zorg thuis, het dienstenpakket, enz.)**

❑ nooit

❑ soms

❑ meestal

❑ altijd

- niet van toepassing/niet nodig

1. **Geven de zorgverleners u voldoende informatie over waar u zorg of hulp kunt krijgen voor uw naaste als zij het niet kunnen geven? (bijvoorbeeld telefoonnummer Welzijn Ouderen)**

❑ nooit

❑ soms

❑ meestal

❑ altijd

- niet van toepassing/niet nodig

1. **Geven de zorgverleners u voldoende informatie over wat er van ú wordt verwacht? (wat u voor uw naaste in huis moet hebben, wat u moet doen, eigen bijdrage, enz.)**

❑ nee

❑ ja

1. **Geven de zorgverleners u voldoende informatie over hoe u uw naaste kunt helpen?**

❑ nooit

❑ soms

❑ meestal

- altijd
- niet van toepassing/niet nodig

1. **Begrijpt u de informatie die u van de zorgverleners krijgt?**

❑ nooit

❑ soms

❑ meestal

- altijd

1. **Kunt u zelf bepalen wat uw rol en taak is in de zorg voor uw naaste?**

❑ nooit

❑ soms

❑ meestal

❑ altijd

- niet van toepassing/niet nodig

###### Communicatie

1. **Weet u waar en bij wie u terecht kunt met uw vragen, problemen en eventuele klachten?**

❑ nooit

❑ soms

❑ meestal

❑ altijd

- niet van toepassing/niet nodig

1. **Heeft u een vaste contactpersoon als aanspreekpunt bij vragen?**

❑ ja

❑ nee

1. **Staan de zorgverleners voldoende open voor uw wensen?**

❑ nooit

❑ soms

❑ meestal

❑ altijd

- niet van toepassing/niet nodig

1. **Vindt u dat de zorgverleners goed reageren op uw vragen en suggesties?**

❑ nooit

❑ soms

❑ meestal

❑ altijd

- niet van toepassing/niet nodig

1. **Overleggen de zorgverleners met u over wat er moet gebeuren?**

❑ nooit

❑ soms

❑ meestal

❑ altijd

- niet van toepassing/niet nodig

1. **Kunt u de zorgverleners telefonisch goed bereiken?**

❑ nooit

❑ soms

❑ meestal

❑ altijd

- weet ik niet/geen ervaring mee

###### Evaluatie zorg

1. **Bespreken de zorgverleners met u of u tevreden bent over de zorg aan uw naaste?**

❑ nooit

❑ 1 keer per jaar

❑ meerdere keren per jaar

- niet van toepassing/niet nodig

1. **Maken de zorgverleners samen met u nieuwe afspraken als uw naaste andere, meer of minder zorg nodig heeft?**

❑ nooit

❑ soms

❑ meestal

❑ altijd

- niet van toepassing/niet nodig

1. **Brengen zorgverleners elkaar op de hoogte van veranderingen in de zorg voor uw naaste of moet u ze dat vertellen?**

❑ de zorgverleners nemen hierover contact met elkaar op

❑ ik bemerk dat ze het niet horen van elkaar, dus ik vertel het

❑ niet van toepassing/weet niet

1. **Als er problemen zijn, kunt u dan voldoende terecht bij de zorgverleners om deze te bespreken?**

❑ nooit

❑ soms

❑ meestal

❑ altijd

- niet van toepassing/niet nodig

###### Professionaliteit

1. **Zijn de zorgverleners beleefd tegen u?**

❑ nooit

❑ soms

❑ meestal

❑ altijd

- niet van toepassing/niet nodig

1. **Hebben de zorgverleners genoeg tijd voor u?**

❑ nooit

❑ soms

❑ meestal

❑ altijd

- niet van toepassing/niet nodig

1. **Luisteren de zorgverleners aandachtig naar u?**

❑ nooit

❑ soms

❑ meestal

❑ altijd

- niet van toepassing/niet nodig

1. **Nemen de zorgverleners u serieus?**

❑ nooit

❑ soms

❑ meestal

- altijd
- niet van toepassing/niet nodig

###### Verzorging en gezondheid

1. **Krijgt u voldoende hulp en begeleiding van de zorgverleners?**

❑ nooit

❑ soms

❑ meestal

❑ altijd

- niet van toepassing/niet nodig

1. **Houden de zorgverleners voldoende rekening met wat u zelf wel en niet kunt?**

❑ nooit

❑ soms

❑ meestal

❑ altijd

- niet van toepassing/niet nodig

1. **Letten of wijzen de zorgverleners op veranderingen in uw gezondheid?**

❑ nooit

❑ soms

❑ meestal

❑ altijd

- niet van toepassing/niet nodig

1. **Hebben de zorgverleners voldoende aandacht voor hoe het met u gaat?**

❑ nooit

❑ soms

❑ meestal

❑ altijd

- niet van toepassing/niet nodig

1. **Bieden de zorgverleners u voldoende emotionele ondersteuning als gesprekspartner of luisterend oor?**

❑ nooit

❑ soms

❑ meestal

❑ altijd

- niet van toepassing/niet nodig

1. **Houden zorgverleners voldoende rekening met uw behoeftes?**

❑ nooit

❑ soms

❑ meestal

❑ altijd

- niet van toepassing/niet nodig

###### Veiligheid

1. **Vertellen de zorgverleners u over mogelijkheden voor woningaanpassingen of hulpmiddelen?**

❑ nee

❑ ja

- niet van toepassing/niet nodig

1. **Hebben de zorgverleners met u afgesproken wat u in geval van nood moet doen? (welk telefoonnummer u kunt bellen, wanneer en hoe u de alarmering gebruikt, enz.)**

❑ nee

❑ ja

- niet van toepassing/niet nodig

1. **Voelt u zich veilig en op uw gemak in aanwezigheid van de zorgverleners?**

❑ nooit

❑ soms

❑ meestal

❑ altijd

- niet van toepassing/niet nodig

1. **Helpen de zorgverleners u voldoende bij het vinden van mogelijkheden voor dagbesteding, sociale contacten en activiteiten voor uzelf?**

❑ nooit

❑ soms

❑ meestal

❑ altijd

- niet van toepassing/niet nodig

###### Wachttijd

1. **Krijgt u van zorgverleners genoeg informatie over de wachttijd voor de zorg aan uw naaste en wat u in de tussentijd kunt doen?**

❑ ruim voldoende

❑ voldoende

❑ onvoldoende

❑ volstrekt onvoldoende

- niet van toepassing (geen wachttijd)

###### Algemene beoordeling zorg en begeleiding aan uzelf

1. **Welk cijfer geeft u de zorgverleners voor de zorg en begeleiding aan uzelf? Een 0 betekent: heel erg slecht. Een 10 betekent: uitstekend.**

- 0 *heel erg slechte zorgverleners*
- 1
- 2
- 3
- 4
- 5
- 6
- 7
- 8
- 9
- 10 *uitstekende zorgverleners*

1. **Wat zou u graag anders willen in de zorg die uw naaste krijgt?**

**67. Wat zou u graag anders willen in de zorg en begeleiding die u zelf krijgt?**

**Hartelijk dank voor het invullen van de vragenlijst!**
