# Supplementary material for: A quasi-experimental study of the effects of an integrated care intervention for the frail elderly on informal caregivers’ satisfaction with care and support
Source: BMC Health Serv Res. 2014 Mar 29;14:140. doi: 10.1186/1472-6963-14-140 (PMC3986650; doi:10.1186/1472-6963-14-140)
Supplement: Additional file 3 — Within- and between-group analysis of mean item scores at T0 and T1. Description of file: Table showing mean scores, standard deviations and significant changes within and between the experimental and control groups. [file 1472-6963-14-140-S3.docx]

Additional File 3. Within- and between-group analysis of mean item scores at T0 and T1

| **Dimensions and items** | | | **Experimental** | | | **Control** | | | **Δ** |
| --- | --- | --- | --- | --- | --- | --- | --- | --- | --- |
| **I/C** | | **Information and Communication** | **T0** (sd) | **T1** (sd) | **Δ** | **T0** (sd) | **T1** (sd) | **Δ** | ***p*** |
| C | | Professionals adhere to arrangements | 3.5 (0.51) | 3.3 (0.54) | -0.19* | 3.5 (0.58) | 3.4 (0.49) | -0.11 | - |
| C | | Use of care plan for information exchange | 3.1 (0.86)# | 3.1 (0.86) | 0.00 | 3.4 (0.59)# | 3.3 (0.64) | -0.13 | - |
| C | | Coordination of professionals (who visits when) | 3.2 (0.83)# | 3.3 (0.65)# | 0.10 | 3.5 (0.52)# | 3.8 (0.41)# | 0.37 | - |
| C | | Time of visits suits CR | 3.2 (0.56) | 3.3 (0.67) | 0.03 | 3.4 (0.58) | 3.3 (0.48) | -0.05 | - |
| C | | Sufficient information about care provided to CR | 3.0 (0.75) | 3.0 (0.69) | 0.00 | 3.1 (0.74) | 3.4 (0.50) | 0.26# | - |
| I | | Sufficient information about care provided to IC | 2.2 (1.07) | 2.6 (1.10) | 0.32 | 2.6 (1.20) | 2.6 (0.96) | 0.00 | - |
| C | | CR understands information | 2.9 (0.74) | 2.8 (0.79) | -0.10 | 3.0 (0.89) | 2.8 (0.87) | -0.19 | - |
| I | | IC understands information | 3.3 (0.78) | 3.2 (1.04)* | -0.15 | 3.6 (0.61) | 3.8 (0.55)* | 0.20 | - |
| I | | Sufficient information about services | 1.8 (1.10) | 2.0 (1.03) | 0.17 | 2.8 (1.48) | 2.6 (1.08) | -0.15 | - |
| I | | Sufficient information about expectations | 2.0 (1.43) | 2.1 (1.46) | 0.09 | 2.3 (1.51) | 2.6 (1.53) | 0.35 | - |
| I | | Sufficient information about how to provide care | 1.8 (0.97) | 2.3 (1.16) | 0.53* | 2.1 (1.14) | 2.1 (1.10) | 0.07 | - |
| I | | IC knows who to call for complaints, problems, emergency | 3.4 (0.88) | 3.3 (0.87)# | -0.07 | 3.3 (0.91) | 3.7 (0.48)# | 0.38* | # |
| I | | Fixed/central source of information | 2.6 (1.52) | 2.8 (1.50) | 0.17 | 2.7 (1.51) | 2.7 (1.51) | 0.00 | - |
| I | | Professionals easily reached by phone | 3.4 (0.85) | 3.4 (0.73) | 0.00 | 3.6 (0.51) | 3.5 (0.61) | -0.11 | - |
| I | | Professionals keep each other informed about care | 3.5 (1.18)* | 3.3 (1.31)* | -0.18 | 4.0 (0.00)* | 4.0 (0.00)* | 0.00 | - |
| C | | Coordination of professionals (who does what) | 3.0 (0.74) | 3.2 (0.71) | 0.16 | 3.3 (0.63) | 3.6 (0.51) | 0.31 | - |
| **Involvement and Control** | | | **T0** (sd) | **T1** (sd) | **Δ** | **T0** (sd) | **T1** (sd) | **Δ** | ***p*** |
| C | | CR is involved in decisions regarding care | 3.4 (0.82) | 3.2 (0.96) | -0.23 | 3.4 (0.88) | 3.3 (0.70) | -0.11 | - |
| I | | IC is involved in decisions regarding care | 2.5 (1.16) | 2.6 (1.08) | 0.09 | 2.8 (1.15) | 2.7 (1.21) | -0.05 | - |
| I | | IC has control over own role and care tasks | 3.4 (0.69) | 3.6 (0.63) | 0.18 | 3.7 (0.64) | 3.5 (0.59) | -0.17 | - |
| I | | Professionals discuss care tasks with IC | 2.2 (1.16) | 2.6 (0.87) | 0.40# | 2.8 (0.97) | 2.8 (0.83) | 0.00 | - |
| I | | If needed, professionals make new care arrangements with IC | 2.1 (1.28) | 2.4 (1.19) | 0.26 | 2.3 (1.29) | 2.8 (1.15) | 0.37# | - |
| **Client-centeredness and Professionalism** | | | **T0** (sd) | **T1** (sd) | **Δ** | **T0** (sd) | **T1** (sd) | **Δ** | ***p*** |
| C | | Professionals are open to needs of CR | 3.5 (0.51) | 3.4 (0.55) | -0.10 | 3.4 (0.58) | 3.4 (0.50) | 0.04 | - |
| I | | Professionals are open to needs of IC | 3.1 (0.79) | 3.2 (0.61) | 0.18 | 3.2 (0.66) | 3.4 (0.62) | 0.17 |  |
| C | | Adequate reaction to questions of CR | 3.5 (0.57)# | 3.4 (0.57) | -0.04 | 3.2 (0.49)# | 3.2 (0.58) | 0.00 | - |
| I | | Adequate reaction to questions of IC | 3.0 (0.73) | 3.1 (0.58) | 0.11 | 3.2 (0.63) | 3.0 (0.58) | -0.21 | - |
| C | | Professionals are polite to CR | 3.7 (0.53)# | 3.7 (0.44) | 0.05 | 3.9 (0.32)# | 3.7 (0.48) | -0.22* | * |
| I | | Professionals are polite to IC | 3.9 (0.34) | 3.8 (0.40) | -0.06 | 3.9 (0.28) | 3.8 (0.42) | -0.13 | - |
| C | | Professionals have sufficient time for CR | 3.0 (0.68) | 3.1 (0.58) | 0.06 | 3.0 (0.74) | 3.2 (0.69) | 0.12 | - |
| I | | Professionals have sufficient time for IC | 2.9 (0.73)* | 3.3 (0.56) | 0.39* | 3.4 (0.71)* | 3.0 (0.79) | -0.41# | * |
| C | | Professionals are informed about health of CR | 3.5 (0.57) | 3.4 (0.66) | -0.15# | 3.5 (0.65) | 3.5 (0.51) | 0.00 | - |
| C | | Professionals handle possessions carefully | 3.6 (0.63) | 3.5 (0.68) | -0.10 | 3.8 (0.40) | 3.6 (0.50) | -0.22* | - |
| I | | IC can turn to professional in case of problems | 3.0 (0.85) | 3.3 (0.71) | 0.22 | 3.3 (0.66) | 3.6 (0.61) | 0.25 | - |
| I | | Professionals listen attentively to IC | 3.1 (0.80) | 3.3 (0.62) | 0.23 | 3.2 (0.71) | 3.4 (0.69) | 0.21 | - |
| I | | Professionals take IC seriously | 3.3 (0.80) | 3.6 (0.65) | 0.24 | 3.5 (0.61) | 3.6 (0.51) | 0.11 | - |
| C | | Professionals take functional abilities of CR into account | 3.4 (0.74) | 3.5 (0.57) | 0.12 | 3.6 (0.58) | 3.5 (0.59) | -0.08 | - |
| I | | Professionals take functional abilities of IC into account | 2.7 (0.79) | 3.2 (0.98) | 0.45# | 3.6 (0.54) | 3.3 (0.49) | -0.28 | * |
| C | | Professionals are attentive to changes in health of CR | 3.4 (0.94) | 3.5 (1.47) | 0.06 | 3.5 (0.67) | 3.6 (0.50) | 0.13 | - |
| I | | Professionals are attentive to changes in health of IC | 1.9 (0.85) | 2.2 (0.77) | 0.27 | 3.2 (1.17) | 2.7 (1.37) | 0.50 | - |
| C | | Professionals pay attention to overall well-being of CR | 3.3 (0.65) | 3.5 (0.73) | 0.17 | 3.5 (0.59) | 3.6 (0.51) | 0.04 | - |
| I | | Professionals pay attention to overall well-being of IC | 2.1 (1.24) | 2.4 (1.31) | 0.34 | 2.6 (1.30) | 2.8 (0.89) | 0.12 | - |
| C | | Professionals provide sufficient emotional support to CR | 2.8 (0.72) | 3.0 (0.72) | 0.18 | 3.1 (0.78) | 2.9 (0.66) | -0.18 | - |
| I | | Professionals provide sufficient emotional support to IC | 2.3 (1.14)# | 2.4 (1.15) | 0.07 | 3.1 (0.84)# | 2.8 (0.89) | 0.62 | - |
| C | | Care is provided according to wishes of CR | 3.5 (0.51) | 3.3 (0.55) | -0.13 | 3.5 (0.51) | 3.1 (0.29) | -0.43*** | * |
| I | | Support is provided according to needs of IC | 2.4 (1.27)# | 2.8 (1.23) | 0.40 | 3.5 (0.84)# | 2.8 (0.98) | -0.67 | # |
| I | | IC feels comfortable/safe around professionals | 3.8 (0.42) | 3.7 (0.46) | -0.08 | 3.7 (0.59) | 3.6 (0.63) | -0.13 | - |
| **Amount and Quality of care** | | | **T0** (sd) | **T1** (sd) | **Δ** | **T0** (sd) | **T1** (sd) | **Δ** | ***p*** |
| C | | Number of professionals visiting | 2.7 (0.64) | 2.9 (0.68) | 0.23 | 2.8 (0.54) | 3.0 (0.50) | 0.19 | - |
| C | | Professionals evaluate care with CR | 3.7 (0.97) | 3.7 (0.97) | 0.00 | 3.4 (1.27) | 3.7 (0.95) | 0.30# | - |
| I | | Professionals evaluate care with IC | 2.3 (1.52) | 2.9 (1.49) | 0.63* | 1.8 (1.37) | 2.4 (1.55) | 0.60 | - |
| C | | Professionals are competent | 3.3 (0.60) | 3.4 (0.61) | 0.09 | 3.5 (0.59) | 3.5 (0.51) | 0.00 | - |
| C | | Professionals collaborate adequately with others | 3.3 (0.74) | 3.3 (0.84) | 0.00 | 3.4 (0.80) | 3.5 (0.62) | 0.06 | - |
| C | | Professionals are attentive to the safety of CR | 2.9 (0.99) | 2.7 (0.87)* | -0.21 | 3.0 (0.97) | 3.1 (0.77)* | 0.15 | - |
| C | | Professionals help to find services and activities for CR | 2.8 (1.25) | 2.5 (1.10) | -0.23 | 2.3 (1.23) | 2.8 (1.12) | 0.50 | * |
| I | | Professionals help to find support services and activities for IC | 2.3 (1.53) | 2.0 (1.73) | -0.33 | 2.4 (1.34) | 2.4 (1.34) | 0.00 | - |
| C | | Professionals provide sufficient help with administrative tasks | 2.0 (0.97) | 1.6 (0.81) | -0.44# | 2.0 (1.21) | 2.3 (1.07) | 0.33 | * |
| C | | Sufficient amount of care for CR | 3.5 (1.15) | 3.7 (0.85) | 0.25 | 3.4 (1.23) | 3.9 (0.60) | 0.48 | - |
| I | | Sufficient amount of support for IC | 2.8 (0.87) | 3.0 (0.89) | 0.18 | 3.3 (1.00) | 3.0 (1.23) | -0.33 | - |
| I | | Professionals provide information about in-home adjustments | 2.7 (1.58) | 2.3 (1.58) | -0.34 | 3.0 (1.50) | 3.3 (1.32) | 0.33 | - |
| I | | Professionals discuss what to do in case of emergency | 2.6 (1.53) | 2.7 (1.52) | 0.13 | 2.9 (1.50) | 3.3 (1.34) | 0.37 | - |
| C | | Acceptability of waiting time for care for CR | 3.0 (0.00) | 3.1 (0.35) | 0.13 | 2.9 (0.73) | 2.9 (0.62) | 0.00 | - |
| I | | Information regarding waiting time is provided | 3.0 (0.00) | 3.0 (0.00) | 0.00 | 3.0 (0.00) | 3.0 (0.00) | 0.00 | - |
| **Overall Satisfaction** | | | **T0** (sd) | **T1** (sd) | **Δ** | **T0** (sd) | **T1** (sd) | **Δ** | ***p*** |
| I | Overall satisfaction with support of IC | | 6.4 (0.90) | 6.5 (0.71) | 0.06 | 6.6 (1.15) | 6.9 (0.93) | 0.31 | - |
| C | Overall satisfaction with care for CR | | 7.6 (2.11) | 7.6 (2.01) | -0.09 | 7.6 (2.10) | 7.6 (1.84) | -0.04 | - |

#p<0.10; *p<0.05; **p<0.01; ***p<0.001

C=Satisfaction with care to care recipient; I=Satisfaction with support of informal caregiver; IC=Informal caregiver; CR=Care receiver;

**Δ**=difference between scores at T0 and T1; Δ group=difference between experimental and control groups in changes between T0 and T1
