# Supplementary material for: A quasi-experimental study of the effects of an integrated care intervention for the frail elderly on informal caregivers’ satisfaction with care and support
Source: BMC Health Serv Res. 2014 Mar 29;14:140. doi: 10.1186/1472-6963-14-140 (PMC3986650; doi:10.1186/1472-6963-14-140)
Supplement: Additional file 4 — Adjusted R2, β and p-values for items regarding satisfaction with care provided to care recipients (CR). Description of file: Results of regression analyses on items regarding care to the care recipient. [file 1472-6963-14-140-S4.docx]

| **Satisfaction with care** | **Adj. R2** | **T0** | **Age** | **Sex** | **Together** | **WICM** |
| --- | --- | --- | --- | --- | --- | --- |
| Professionals use care plan | 18% | **.317*** | .286 | .073 | .074 | .012 |
| Professionals coordinate visits | 26% | **.298#** | -.100 | -.356 | .092 | -.263 |
| Professionals adhere to arrangements | 25% | **.415**** | **.379*** | -.113 | -.232 | -.024 |
| Time of visits is suitable | n.s. | **-** | - | - | - | - |
| Professionals involve care recipient | 19% | **.509***** | .060 | .076 | .045 | -.082 |
| Professionals provide sufficient information | 22% | **.381*** | .236 | -.102 | .057 | -.137 |
| Care recipient understands information | 28% | **.470***** | **.299#** | **.281#** | .087 | .065 |
| Professionals evaluate care process frequently | n.s. | - | - | - | - | - |
| Professionals coordinate care tasks | n.s. | - | - | - | - | - |
| Professionals respond adequately to questions | n.s. | - | - | - | - | - |
| Professionals are attentive to needs | n.s. | - | - | - | - | - |
| Professionals are polite | n.s. | - | - | - | - | - |
| Professionals have sufficient time | 23% | **.382**** | **.455**** | -.065 | **-.274#** | .034 |
| Professionals are informed about health | 15% | **.386**** | .164 | -.155 | -.157 | -.065 |
| Professionals are careful with belongings | 13% | **.289*** | **.331#** | .020 | -.036 | .025 |
| Professionals collaborate well with others | 26% | **.514**** | .099 | -.018 | .088 | -.032 |
| Professionals provide good quality of care | 30% | **.430**** | .108 | **-.301*** | -.050 | .030 |
| Professionals are attentive to changes in health | 23% | **.487***** | .040 | -.091 | .163 | -.048 |
| Professionals are attentive to overall well-being | 26% | **.453**** | **.397*** | -.088 | **-.305#** | .092 |
| Professionals take functional ability into account | 24% | **.482***** | .206 | -.155 | -.047 | .141 |
| Professionals provide care according to wishes | 30% | **.370**** | .135 | **-.293*** | -.123 | **.376**** |
| Professionals help with administrative affairs | 37% | **.580**** | -.249 | .065 | .273 | **-.452*** |
| Professionals pay sufficient attention to safety | 36% | **.441**** | **.393*** | -.018 | -.213 | -.185 |
| Professionals help in finding activities | 47% | **.557**** | .118 | **-.355*** | .108 | -.082 |
| Professionals provide sufficient emotional supp | 19% | **.333*** | .311 | -.200 | -.143 | .178 |
| The care recipient receives sufficient care | n.s. | - | - | - | - | - |
| Number of professionals is sufficient | n.s. | - | - | - | - | - |
| Acceptability of waiting time | n.s. | - | - | - | - | - |
| Overall satisfaction with care | 29% | **.534***** | **.413**** | .138 | -.179 | .026 |

Additional File 4. Adjusted R2, β and *p*-values for items regarding satisfaction with care provided to care recipients (CR)

#p<0.10; *p<0.05: **p<0.01; ***p<0.001 (shown in bold)

n.s.=model not significant
