# Supplementary material for: A quasi-experimental study of the effects of an integrated care intervention for the frail elderly on informal caregivers’ satisfaction with care and support
Source: BMC Health Serv Res. 2014 Mar 29;14:140. doi: 10.1186/1472-6963-14-140 (PMC3986650; doi:10.1186/1472-6963-14-140)
Supplement: Additional file 5 — Adjusted R2, β and p-values for items regarding satisfaction with support of informal caregivers (IC). Description of file: Results of regression analyses on items regarding care to the informal caregiver. [file 1472-6963-14-140-S5.docx]

Additional File 5. Adjusted R2, β and *p*-values for items regarding satisfaction with support of informal caregivers (IC)

| **Satisfaction with support** | **Adj. R2** | **T0** | **Age** | **Gender** | **Together** | **WICM** |
| --- | --- | --- | --- | --- | --- | --- |
| Professionals provide sufficient information: care | 42% | **.578***** | .035 | -.136 | .195 | .137 |
| Professionals provide sufficient information: services | 40% | **.576**** | .034 | -.240 | .020 | .028 |
| Professionals provide sufficient information: expect | 39% | **.459***** | -.005 | -.190 | **.217#** | -.094 |
| Professionals provide sufficient information: how | 43% | **.311#** | .060 | -.073 | **.461*** | .165 |
| Understands information | 23% | **.442**** | -.101 | -.029 | .195 | **-.241#** |
| Arrangements for emergency situations | 27% | **.406*** | .113 | -.129 | .108 | -.075 |
| Professionals discusses tasks with caregiver | 23% | **.429**** | -.010 | .030 | **.310#** | .053 |
| Being sufficiently involved in care decisions | 57% | **.591***** | -.054 | .136 | **.437**** | .036 |
| Availability of professionals in case of problems | n.s. | - | - | - | - | - |
| Professionals make arrangements if care changes | 46% | **.661***** | -.037 | .031 | .149 | -.132 |
| Professionals react adequately | n.s. | - | - | - | - | - |
| Professionals take me seriously | n.s. | - | - | - | - | - |
| Professionals have sufficient time | n.s. | - | - | - | - | - |
| Professionals listen carefully | 14% | **.276#** | .185 | -.276 | -.059 | -.008 |
| Professionals are polite | n.s. | - | - | - | - | - |
| Feels comfortable/safe around professionals | n.s. | - | - | - | - | - |
| Professionals are easily accessible by phone | 22% | **.313*** | **.514*** | .132 | -.045 | .063 |
| One professional /contact point | 19% | **.450***** | .037 | -.002 | .138 | .045 |
| Knows where/whom to go with complaints | 29% | **.362**** | **.272#** | -.197 | .016 | **-.198#** |
| Information is provided about waiting time | n.s. | - | - | - | - | - |
| Professionals keep each other informed | 66% | **.809***** | .100 | .006 | -.023 | -.069 |
| Professionals take needs into account | n.s. | - | - | - | - | - |
| Sufficient assistance and support is provided | 68% | **.730***** | **.346#** | -.053 | .135 | .233 |
| Professionals pay attention to changes is health | 69% | **.617**** | **.706*** | .273 | .100 | .193 |
| Professionals pay sufficient attention to well-being | 75% | **.545**** | .278 | -.028 | .333 | .062 |
| Professionals provide sufficient emotional support | n.s. | - | - | - | - | - |
| Professionals take functional abilities into account | 45% | **.647*** | **.529#** | .269 | .223 | .353 |
| Professionals are open to wishes | n.s. | - | - | - | - | - |
| Professionals provide information in-home adjustments | 62% | **.690**** | .250 | -.085 | -.022 | -.220 |
| Professionals help find support services and activities | n.s. | - | - | - | - | - |
| Professional evaluate care with caregiver | 38% | **.568**** | .186 | .129 | .084 | .105 |

#p<0.10; *p<0.05: **p<0.01; ***p<0.001 (shown in bold)

n.s.=model not significant
